# Respiratory microbiomes reflect whale health

**DOI:** 10.1093/ismejo/wraf231

**Published:** 2025-11-12

**Authors:** Carolyn A Miller, Enrico Pirotta, Sharon Grim, Michael J Moore, John W Durban, Peter L Tyack, Holly Fearnbach, Samantha G M Leander, Amy R Knowlton, Amy M Warren, Monica A Zani, Regina Asmutis-Silvia, Heather M Pettis, Amy Apprill

**Affiliations:** Woods Hole Oceanographic Institution, Woods Hole, MA 02543, United States; Centre for Research into Ecological and Environmental Modelling, University of St Andrews, St Andrews KY16 9LZ, United Kingdom; Woods Hole Oceanographic Institution, Woods Hole, MA 02543, United States; Woods Hole Oceanographic Institution, Woods Hole, MA 02543, United States; Anderson Cabot Center for Ocean Life, New England Aquarium, Boston, MA 02110, United States; Sea Mammal Research Unit, School of Biology Scottish Oceans Institute, University of St Andrews, St Andrews KY16 8LB, United Kingdom; SR^3^, SeaLife Response, Rehabilitation and Research, Des Moines, WA 98198, United States; SR^3^, SeaLife Response, Rehabilitation and Research, Des Moines, WA 98198, United States; Anderson Cabot Center for Ocean Life, New England Aquarium, Boston, MA 02110, United States; Anderson Cabot Center for Ocean Life, New England Aquarium, Boston, MA 02110, United States; Anderson Cabot Center for Ocean Life, New England Aquarium, Boston, MA 02110, United States; Whale and Dolphin Conservation, Plymouth, MA 02360, United States; Anderson Cabot Center for Ocean Life, New England Aquarium, Boston, MA 02110, United States; Woods Hole Oceanographic Institution, Woods Hole, MA 02543, United States

**Keywords:** wildlife health assessment, North Atlantic right whale, drone, photogrammetry, body condition, microbiome, bacteria, elastic net regression

## Abstract

As important members of the marine ecosystem, baleen whales are frequently managed and protected, but methodology to assess their health remains limited. Recent technological advances, such as the use of drones, support the non-invasive collection of promising health-associated data, including respiratory exhalant microbiota. Here, we considered five health metrics paired with respiratory exhalant samples to examine the utility of characterizing respiratory microorganisms for health diagnostics of North Atlantic right whales (*Eubalaena glacialis*), one of the most endangered baleen whale species. In 2016–2024, we used drones to collect 103 exhalant samples from 85 individuals to examine the associated microbiome, using amplicon sequencing methods targeting bacteria and archaea. The health status of sampled whales was characterized using an index of body condition derived from full-body vertical drone images, three qualitative assessments obtained from photo-identification imagery, and an existing health and vital rates model. Using an elastic net penalized regression approach, we demonstrate significant relationships between these health metrics and respiratory-associated microorganisms. Bacterial taxa that significantly contributed to the model for the body condition index differed between the thinnest and most robust males in the dataset. The thin whale harbored taxa belonging to the same genus as mammalian pathogens, *Clostridium* and *Peptoniphilus*, whereas the robust whale harbored taxa commonly observed in lipid-rich environments, *Sediminispirochaeta* and *Candidatus* Gracilibacteria. These differences warrant further investigation into the mechanisms by which bacteria contribute to whale health. Our findings demonstrate the utility of non-invasive multi-metric health models that include respiratory exhalant microbiota for whale health assessment and management.

Quantifying the health of wildlife is important for status assessments and management of populations that are increasingly threatened by the cumulative risk posed by sublethal effects of increasing anthropogenic stressors. Microorganisms play a key role in animal health and disease, yet knowledge of microbial associates is lacking for most wildlife species, especially whales, due to their large size and high mobility within the ocean. Drone-based collection and subsequent identification of respiratory exhalant microorganisms was recently used to show microbiota consistency and variability in whales [1, 2]. However, the respiratory microbiomes of free ranging whales have not been examined for any individuals with a described health state, thus the utility of characterizing microorganisms for whale health diagnostics remains unknown.

North Atlantic right whales (*Eubalaena glacialis*; NARW) present an ideal case study to examine if and how the respiratory exhalant microbiome reflects whale health. Following centuries of intensive whaling, the species was recognized as critically depleted and nearing extinction as early as the 1930s, which provided motivation for scientists and management agencies to monitor the life history and health of each whale remaining in the population (currently <400) [3]. NARW health status varies widely, ranging from emaciated individuals with skin lesions, rake marks, and cyamid ectoparasites, to relatively robust individuals appearing in good overall health [4].

Here, we collated multi-metric health data for 85 distinct NARW from which 103 respiratory exhalant samples were collected in Cape Cod Bay, Massachusetts, USA, during the spring feeding seasons 2016–2024. We analyzed five metrics of individual health status: a body condition index derived from vertical drone-based images using photogrammetric techniques [5], an estimate of overall individual health from a model for NARW survival and calving probability [6, 7], and three categorical variables from a visual health assessment (VHA) [8]. Respiratory exhalant microbiota were examined in drone-captured samples using small subunit ribosomal RNA gene partial sequencing methodology, with amplicon sequence variants (ASVs) employed to differentiate distinct microbial taxa, often at single nucleotide resolution (Supplementary Information).

To examine the relationships of the five health metrics, animal age, sex, and sampling year with the respiratory microbiota, we applied elastic net regression analysis to a variable subset of the relative abundance of ASVs selected using a pre-screening procedure (Table S1). Cross-validation suggested the final models resulted in small to moderate median absolute error (MAE) and good predictive power (Table S1). Of the five health metrics, the best performing model was for the modeled health metric [6, 7] (*R*^2^ = 0.82; MAE = 0.1; Fig. 1A); the model’s predictive power held when health estimates and sample collection were offset in time, suggesting the respiratory microbiome may act as an indicator of long-term health status. ASV relative abundances also supported reasonable predictions of the body condition index (Fig. 1B), and 39 ASVs were shared with the elastic net for the modeled health metric. Similarly, respiratory microbiome was associated with VHA scores (Fig. 1C–E), although the sample size for the VHA models was smaller (Table S1). In addition to the associations with health, model results highlighted that the respiratory microbiome was predictive of sampling year, with more precise predictions in recent years when sample size was also larger (Fig. 1F). This result was supported by the elastic net for year when excluding seawater-prevalent ASVs (Fig. S1), suggesting that this connection to year is not related to seawater microbial changes. The model for age showed the poorest predictive power, with an MAE of several years, but observed and predicted ages were nonetheless moderately correlated (Fig. 1G). Finally, respiratory microbiome was found to vary between non-calf females and males; the elastic net performed better at predicting sex for males, suggesting that microbiome differences in females may be influenced by reproductive status (Fig. 1H).

**Figure 1:**
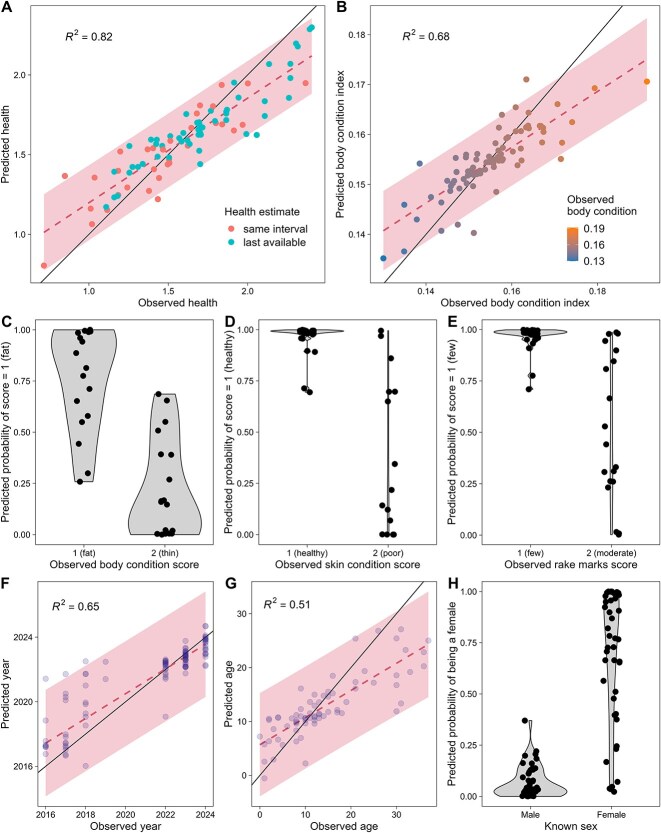
Results of the elastic net regressions for each response variable: (A) modeled health metric, (B) body condition index from photogrammetry, (C) body condition score from visual health assessment (VHA), (D) skin condition score from VHA, (E) rake marks score from VHA, (F) sampling year, (G) age, and (H) sex. Predictions were obtained using leave-one-out cross-validation (see Supplementary Information for an investigation of the influence of individual replicate sampling using leave-one-individual-out cross-validation). In (A), (B), (F), and (G), the dashed line and shaded area indicate the estimated relationship (mean and 95% prediction interval) between observations and elastic net predictions, whereas the black line represents the 1–1 diagonal; the *R*^2^ of the relationship is reported in the top left of each panel. In A, points are colored by whether the health metric was estimated in the same three-month interval as the blow sample collection, March-May, (same interval) or the last available value for an individual was used (last available; Supplementary Information), and in (B), by the observed body condition index. In (C)–(E), violin plots showing how the binary model’s predicted probabilities of being in better health (score = 1) are distributed for individuals who were observed in better health (score = 1) and those who were in worse health (score = 2). In (H), violin plot showing the predicted probabilities of being a female from the elastic net for known male and female individuals.

We also investigated relationships between the health metrics and a set of summary microbial diversity metrics, rather than the relative abundance of individual ASVs. This analysis did not reveal strong correlations (Supplementary Information).

We further examined the microbial taxa retained by the elastic net model for body condition index from photogrammetry by exploring their relative abundance in two adult males: the thinnest (Fig. 2A) and the most robust (Fig. 2B) in the dataset, sampled one year apart. This comparison revealed generally host-associated taxa, albeit represented at low relative abundance, including four ASVs present only in the thin whale’s exhalant microbiota, 11 only in the robust whale’s microbiota, and 2 (*Bradymonabacteria* and *Guggenheimella*) associated with both individuals (Fig. 2C). The thin whale hosted *Clostridum* and *Peptoniphilus*, bacteria that can cause mammalian infections [9, 10], *Psychrobacter*, found in cetacean respiratory exhalant, blowholes or mouths [1, 11], and *Ruegeria*, a common marine heterotroph [12, 13]. The robust whale’s exhalant included microbial taxa that may be indicative of circulating diet-derived fat (Fig. 2C): two *Sediminispirochaeta* bacteria identified in lipid-rich environments [14] and three *Candidatus* Gracilibacteria phyla (BD1–5) that rely on externally derived fatty acids due to a lack of biosynthetic pathways [15]. The robust whale also harbored marine-mammal-associated *Phocoenobacter*, *Tenacibaculum*, and *Oceanivirga* bacteria [16–18], and ASVs associated with *Candidatus* Tenderia, *Zoogloea*, and *Lachnospirales.* The role of these specific bacteria in whale health is speculative and requires research.

**Figure 2:**
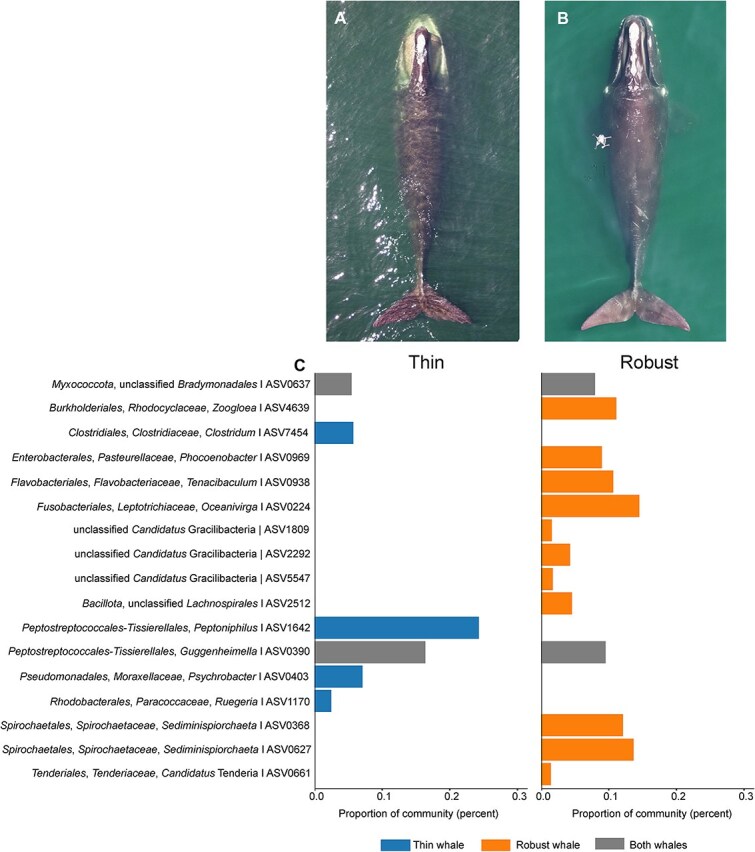
Representative vertical drone images of thin (A) and relatively robust (B) North Atlantic right whales taken from an altitude of ~35 m. (C) The relative abundance of bacterial taxa significantly contributing to the elastic net model for the body condition index differs in the corresponding thin and robust whales in the photographs. Taxa only present in the thin whale are colored in blue, taxa only present in the robust whale in orange, and taxa present in both whales in gray. The exhalant sampling drone is shown in (B), with sampling drone at an approximate altitude of 2–2.5 m.

We identified relevant correlations between NARW exhalant microbiota and several metrics of individual health. These results suggest that respiratory exhalant microbiota could potentially be used as a biomarker of individual health status; however, these correlations require validation using a larger dataset, which may also grant greater accuracy and precision of health predictions. In particular, a larger sample size would support the development of a model for assessing health that accounts for the effect of contextual variables (e.g., age, sex, individual differences, and sampling year) on respiratory microbiota. These findings provide motivation to further explore the specific role of microorganisms in whale health. With the cumulative, sublethal effects of multiple anthropogenic stressors threatening the viability of baleen whales and other wildlife populations [19], understanding factors that contribute to whale health and identifying suitable health indicators, including their microbiota, is important to their management and longevity.

## Supplementary Material

CL_NARW_health_microbiome_Suppl_materials_Oct9resub4_wraf231

Supplementary_Table_3_wraf231

## Data Availability

Data are available in the NCBI SRA under PRJNA1273885 and ENA PRJEB95144.
