# Supplementary material for: Respiratory microbiomes reflect whale health
Source: ISME J. 2025 Nov 12;19(1):wraf231. doi: 10.1093/ismejo/wraf231 (PMC12599319; doi:10.1093/ismejo/wraf231)
Supplement: CL_NARW_health_microbiome_Suppl_materials_Oct9resub4_wraf231 [file cl_narw_health_microbiome_suppl_materials_oct9resub4_wraf231.docx]

**SUPPLEMENTARY INFORMATION**

**Respiratory microbiomes reflect whale health**

Carolyn A. Miller^1^*^, Enrico Pirotta^2^*^, Sharon Grim^1^, Michael J. Moore^1^, John W. Durban^3^, Peter L. Tyack^4^, Holly Fearnbach^5^, Samantha G. M. Leander^5^, Amy R. Knowlton^3^, Amy M. Warren^3^, Monica A. Zani^3^, Regina Asmutis-Silvia^6^, Heather M. Pettis^3^, Amy Apprill^1*^

**Affiliations**

^1^Woods Hole Oceanographic Institution, Woods Hole MA 02540 USA

^2^Centre for Research into Ecological and Environmental Modelling, University of St. Andrews, St Andrews, Fife, UK

^3^Anderson Cabot Center for Ocean Life, New England Aquarium, Boston, MA, USA

^4^Sea Mammal Research Unit, School of Biology, Scottish Oceans Institute, University of St. Andrews, St Andrews, Fife, UK

^5^SR^3^, SeaLife Response, Rehabilitation and Research, Des Moines, Washington, USA

^6^Whale & Dolphin Conservation, Plymouth, MA, USA

^equal contributions

*authors for correspondence,

Amy Apprill, Department of Marine Chemistry and Geochemistry, Woods Hole Oceanographic Institution, 266 Woods Hole Road, MS #4, Woods Hole MA 02543 USA, [aapprill@whoi.edu](mailto:aapprill@whoi.edu)

Carolyn A. Miller, Department of Marine Chemistry and Geochemistry, Woods Hole Oceanographic Institution, 266 Woods Hole Road, MS #4, Woods Hole MA 02543 USA [cmiller@whoi.edu](mailto:cmiller@whoi.edu)

Enrico Pirotta, Centre for Research into Ecological and Environmental Modelling, The Observatory, Buchanan Gardens, St. Andrews, Scotland, KY16 9LZ, UK,

ep343@st-andrews.ac.uk

**Supplementary Methods**

We collated multi-metric health data for North Atlantic right whales (NARW) from which respiratory exhalant samples were collected in Cape Cod Bay, Massachusetts, USA, during the spring feeding seasons (March to May) 2016 – 2024 (Supplementary Table 2). Specifically, we analysed five metrics that capture the health status of sampled NARW: a body condition index derived from vertical drone-based images using photogrammetric techniques [1, 2], an estimate of overall individual health from a model for NARW survival and calving probability [3, 4], and three categorical variables from a visual health assessment (VHA) [5]. Respiratory exhalant microbiota was also examined from drone-based captured samples, using small subunit ribosomal RNA gene partial (amplicon) sequencing methodology.

***Health metrics***

The body condition index was derived from vertical aerial images collected from directly above whales using a remotely controlled drone at altitudes of 30 - 60 m (median = 48 m), that was equipped with a digital Olympus camera with micro 4/3 sensor and a 25 mm lens [full methodological details in 6]. Only images when a whale was fully visible and appeared to be in flat orientation parallel to the water surface were used. Body condition index was calculated as a ratio of pixel measurements of body width at 60% of body length divided by body length, representing a standardised metric of an individual’s energetic status [2]. Pixel resolution at water level was 0.76 cm (range 0.46 cm to 0.90 cm). When available, we used an individual’s body condition index on the same date as a blow sample collection (*n* = 71); otherwise, we used the closest body condition measurement within the same season as the blow sample (varying between 52 days before and 14 days after blow sample collection).

The overall health metric was derived from a previously presented model [3, 4]. This is a Bayesian state-space model for the survival and calving probability of individual whales as a function of latent health at a 3-month scale (Dec-Feb, Mar-May, Jun-Aug, Sep-Nov), covering the period 1970-2019. Specifically, health is a transformed version of survival probability that varies over time and depends on past health status and a series of intrinsic and extrinsic stressors. The model is informed using individual sightings, health scores from the VHA [5, 7], information on sex, age class, calving events, and deaths, and records of anthropogenic traumas (North Atlantic Right Whale Consortium; www.narwc.org/narwc-databases.html). When available (*n* = 33), the health metric in the same interval as the collection of the blow sample was used (all samples were collected in the Mar-May interval); otherwise, the last estimate available for an individual was used for blow samples collected after 2019.

Finally, we considered the raw scores from the VHA [5]. In summary, four categorical variables are scored based on batched photographs of an individual taken within a season/year along an ordinal scale: body condition and the presence of rake marks forward of the blowholes are scored as one of three categories (poor, fair, or good), whereas the presence of cyamids around the blowholes and skin condition are scored using two categories (poor or good). Here, we focused on three of these variables (body condition, skin condition, and rake marks), since only five individuals in our dataset had poor cyamid score. Individuals in our dataset were only scored as fair or good for body condition and rake marks, so all three variables were treated as binary in the analysis. VHA variables are scored from a set of photographs covering a time interval of variable duration (median: 60 days; range: 29-119 days) [5]; when available, each blow sample was associated with the scores in the interval that contained the collection date. VHA data were only available until the end of 2022 [7].

***Exhalant microbiomes***

We sterilized the drone surfaces with 95% isopropanol and then flew it 2 - 2.5 m over the surfacing whale using a pilot and co-pilot, which allowed the team to track the whale using live video feed from the drone and position the drone above the blowhole so that it could capture exhalant breath condensate (blow) from the whale when it surfaced. Each blow was captured on a new sterile 150-mm petri dish mounted on top of the drone, away from downdraft from the propellers. After the flight, the blow was swabbed with flocked swabs (Puritan Medical Products, Guilford, ME, USA). The swabs containing the sample were immediately placed in cryovials and frozen in dry shipper vapors until they could be transferred to a -80 °C freezer at the end of each day. Seawater samples were collected for comparison with the blow microbiomes. These surface seawater samples (60 mL or 1 L) were collected in the general area where blow sampling was conducted. The seawater was filtered for microbial biomass via peristalsis or syringe using a 25 mm filter holder (Swinnex-25, Millipore Corporation, Burlington, MA, USA) with a 0.2 μm Supor filter (Pall, Port Washington, New York, USA). Technical controls were collected, processed, and sequenced to assess sources of contamination; these included flight controls, i.e., flying the drone with a sterile petri dish with no whales in the area and subsequently swabbing the dish for aerosol-associated microbes; processing controls, i.e., blanks processed in the DNA extraction process; and PCR controls, i.e., sterile water used in the PCR process.

Samples were processed to produce partial sequence reads of associated bacteria and archaea 16S rRNA genes using methods as previously outlined [8] with 515FY and 806RB primers [9, 10] and MiSeq System (Illumina, San Diego CA USA) paired end (250 bp) sequencing (NCBI SRA BioProject: PRJNA1273885). Sequences were quality filtered and bacterial and archaeal taxa differentiated into exact sequence variants called Amplicon Sequence Variants (ASVs) using the DADA2 bioinformatics pipeline [11]. The dataset was reviewed to remove low-quality samples and replicates were either averaged or removed. Contaminant sequences were statistically identified using the decontam R-package [12] and subsequently removed. For each ASV, the decontam software models the likelihood of it being a contaminant in biological samples, based on either or a combination of 1) the prevalence of that ASV in biological samples compared to control samples, and 2) the frequency of that ASV appearing in biological samples compared to control samples. We analyzed 103 samples of blow collected from 85 different North Atlantic right whales between 2016-2024, 33 seawater controls, 27 drone flight controls, and 31 laboratory controls for microbial (bacterial and archaeal) community composition. With two types of biological samples (whale exhalant and seawater), and two experimental control types, it is reasonable to expect that the decontam model would not straightforwardly identify contaminant ASVs across the entire dataset. The taxonomies, relative abundances, and distributions with respect to sample type of ASVs the decontam model flagged as putative contaminants were examined. Using that combination approach, 190 ASVs were considered potential contaminants and removed from the dataset (Supplementary Table 3). Analyses of the six million sequences from the 103 blow samples indicated 6,053 microbial taxa present in the whale dataset, with an average ± 1 SD of 228 ± 81 microbial taxa per sample.

***Statistical analysis***

*Response and explanatory variables*

We investigated the relationships between blow microbiome and the five response variables representing the health status of sampled individuals. We also explored the association of blow microbiome with the age of sampled whales, derived from the NARW Catalog (<http://rwcatalog.neaq.org>) and available for individuals that were first sighted as calves, the sex of sampled whales, also derived from the NARW Catalog, and the sampling year. The analysis of body condition index, modelled health metric, sex, and VHA categorical scores was restricted to blow samples collected from non-calf individuals of known identity (i.e., recorded in the NARW Catalog), and any sample that was potentially a mixture of the blow from a female and her calf was excluded. However, samples from known calves were included in the analysis of age, and samples from known calves and potential female/calf mixture samples were included in the analysis of sampling year. Body condition and respiratory microbiota can change rapidly over time. As a conservative cut-off, we used seven days between samples from the same individual; thus, if a whale was re-sampled after seven days’ time, both samples were included in the analysis, otherwise only one of the repeat samples was included (the mean ± SD Bray-Curtis dissimilarity for samples from the seven whales sampled twice in a season more than seven days apart was 0.58 ± 0.09, whereas the mean for all samples, excluding the repeat samples, was 0.67 ± 0.12). The resulting sample sizes for each analysis are summarized in Supplementary Table 2.

Microbiome data were included in the analysis either as the relative abundance of each ASV in a sample (the number of sequences in each ASV divided by the total number of sequences in a sample), or summarized as a series of diversity and other microbiome metrics: richness, inverse Simpson index, beta dispersion, number of pathogen relatives [described in 8], the ratio of pathogen relatives, 80% abundance coverage, the 80% abundance coverage ratio, and the sum of relative abundances of core taxa. Richness was the number of ASVs observed in a sample. The inverse Simpson index is a measure of alpha diversity that accounts for both richness and evenness in a community. Beta dispersion was based on the Bray-Curtis dissimilarity metric and describes the variability in the composition of ASVs across samples. The number of pathogen relatives was the number of ASVs that match to a list of pathogens [described in 8] at the genus level. The ratio of pathogen relatives was the number of pathogen relatives divided by total number of ASVs in a sample. The 80% abundance coverage was the number of ASVs that comprise 80% of the relative abundance in a sample. The 80% abundance coverage ratio was the 80% abundance coverage divided by the total number of ASVs in a sample. The sum of relative abundances of core taxa was the total relative abundance of ASVs detected in 90% of the samples in the dataset.

*Analysis using ASV relative abundance*

We used an elastic net penalized linear regression approach [13] for the analysis of each response variable as a function of ASV relative abundances, fitted using the glmnet package [14] for R [15]. A binomial distribution was used for the analysis of the binary VHA scores and of sex, whereas a Gaussian error distribution was assumed for all other response variables. We investigated a logarithmic transformation of age, but it did not improve the results of the corresponding model.

The elastic net approach performs both regularization and feature selection through a combination of the ridge and LASSO methods and is therefore useful for datasets comprising a small number of samples compared to a large number of potentially collinear predictors. The elastic net assumes that the relationships between ASV relative abundance and a response variable are linear on the scale of the linear predictor, and that each ASV contributes additively. Elastic net regularization requires the specification of two parameters, *α* and *λ*. The mixing parameter, *α*, controls the balance between ridge and LASSO penalties and was set to a value of 0.5. The regularization parameter, *λ*, was optimized for each model via leave-one-out cross-validation.

The elastic net performs variable selection by design, but previous work has shown that pre-screening of putative explanatory variables before inclusion in the model can improve model accuracy [16]. Here, we followed the Iterative Sure Independence Screening (ISIS) approach [16], building on its implementation for the elastic net [17]. Specifically, an elastic net regression was first fitted using a subset of ASVs that showed a Pearson correlation with the response variable greater than a set threshold, *p_s_*. Then, each of the ASVs that were excluded in the first step was included in a generalized linear model with all other retained ASVs. Candidate ASVs were ranked according to their estimated coefficients (representing their marginal correlation with the response) and the top ones were retained, up to a pre-defined maximum number of predictors, *n_s_*. This procedure was repeated iteratively until *n_s_* was reached. The goal of ISIS is thus both to limit the initial exploration to the subset of most highly correlated predictors, as well as to rescue variables that may have been missed in the regularization. We performed a grid search to identify the optimal values of *p_s_* (between 0.1 and 0.4, in 0.05 intervals) and *n_s_* (50, 100, 200, 300, 400, 500, or 600) for each response variable (Supplementary Table 1). Specifically, we selected the values of the two parameters that minimized the residual sum of squares (i.e., the discrepancy between model estimates and observations).

Leave-one-out cross-validation was used to estimate the prediction error of each model: the model was fit to all but one sample and used to predict the response variable of interest using the ASV relative abundances in the sample that was left out. For continuous responses (body condition index, modelled health metric, year, and age), predictive power was summarized as median absolute error (MAE) and the *R*^2^ of a linear regression between observed and predicted values of the response variables; for the binary VHA scores, predictive power was represented as MAE and the distribution of the predicted probability of observing a score of 1 given the observed score; for sex, predictive power was represented as MAE and the distribution of the predicted probability of being a female given the known sex of the sampled whale. These summaries reflected a model’s ability to predict a given response in a new blow sample.

*Excluding seawater-relevant taxa*

The model for sampling year suggested that blow microbiomes varied predictably over time (see *Results*). To investigate whether such variation could be ascribed to the contamination of whale respiratory samples with seawater taxa, we re-ran the elastic net regression for sampling year as the response variable after removing ASVs that were prevalent in seawater samples. Seawater samples were not always collected contemporarily with blow samples, and the number of seawater samples collected each year also varied. To accommodate this technical and temporal heterogeneity, seawater dominant ASVs were determined year-by-year as well as averaging across the study. Seawater sample sequences for each temporal approach were summed to calculate ASV relative abundances for an averaged seawater profile. ASVs were sorted by rank abundance, and the ASVs that cumulatively contributed to at least 80% of the observed sequences (“80% abundance coverage”) in the averaged seawater profile were considered ‘prevalent’ in the seawater microbiome for that time interval. In the six years with seawater sample collection (2016, 2017, 2018, 2022, 2023, 2024), nine ASVs were consistently observed in the seawater-prevalent microbiomes, and the constituents varied between 19 ASVs and 51 ASVs. When averaging all sequenced seawater samples irrespective of collection year, the seawater-prevalent microbiome consisted of 48 ASVs.

*Using leave-one-individual-out cross-validation to estimate prediction error*

Across elastic net models, we treated all samples as independent, including the few instances when multiple samples were collected from the same individual on separate sampling occasions of seven days or more apart. This decision was based on the expectation that the respiratory microbiome would vary more with health status and environmental conditions than due to differences among individuals. In practice, this implied that the cross-validation procedure that we used to estimate the prediction error of each model focused on single samples, rather than on groups of samples from a given individual.

Here, we investigated the influence of this assumption on the results, using one of the health metrics as an example. Specifically, we re-ran the elastic net model for body condition index from photogrammetry using a leave-one-individual-out cross-validation, i.e., excluding all samples from one individual at each fold. Among the 67 distinct individuals included in this analysis, 56 were sampled once, eight were sampled twice, and three were sampled three times.

The modelling approach was re-run twice: in the first run, we repeated the pre-screening procedure as well as the final cross-validation to estimate prediction error; in the second run, only the final cross-validation was repeated, using the same initial set of ASVs as in the analysis reported in the main text. The different cross-validation approach led to a slightly larger prediction interval when the pre-screening of initial ASVs was also repeated (Supplementary Fig. 2a). However, we found that this difference was mainly attributable to the different initial set of ASVs selected by the pre-screening procedure: when the leave-one-individual-out cross-validation was applied using the same set of initial ASVs, model results were effectively indistinguishable from the results obtained using a leave-one-sample-out cross-validation (Supplementary Fig. 2b). Together, these results reiterate the influence played by the pre-screening of ASVs to include in the elastic net, which warrants future exploration using a larger dataset.

*Analysis using diversity metrics*

We first tested the univariate association between each diversity summary metric and each response variable using either a binomial (for the VHA variables) or a Gaussian (for all other response variables) generalized linear model. For each response, we then fitted an elastic net regression that included all diversity metrics. The diversity metrics were scaled by subtracting the mean and dividing by the standard deviation to facilitate convergence. Model fitting followed the procedure described above, but no pre-screening was needed given the small number of covariates.

ASV richness was negatively associated with body condition index (*t* = -2.098; *p* = 0.039) (Supplementary Fig. 3). All other associations between the diversity metrics and the response variables were not significant to the 0.05 significance level. The elastic net models including all diversity metrics did not retain any predictor across all response variables, except in the model for year, where beta dispersion and 80% abundance coverage ratio were retained; however, the *R*^2^ of this model was low (0.06) and the MAE was high (2.7 years).

**SUPPLEMENTARY TABLES**

**Supplementary Table 1.** Sample size, number of distinct individuals, pre-screening parameters (*p_s_* and *n_s_*), number of microbial respiratory exhalant-related ASVs retained after regularization, median absolute error (MAE), and *R*^2^ (predictions vs. observations, for continuous variables only) for the elastic net regression of each response variable as a function of ASV relative abundances.

| **Response variable** | **Sample size** | **Distinct individuals** | **Optimal *p_s_*** | **Optimal *n_s_*** | **Retained ASVs** | **MAE** | ***R*^2^** |
| --- | --- | --- | --- | --- | --- | --- | --- |
| *Body condition index* | 81 | 67 | 0.20 | 600 | 137 | 0.003 | 0.68 |
| *Modelled health metric* | 85 | 68 | 0.10 | 500 | 164 | 0.096 | 0.82 |
| *Body condition (VHA)* | 36 | 32 | 0.20 | 200 | 112 | 0.162 | - |
| *Skin condition (VHA)* | 50 | 43 | 0.35 | 300 | 147 | 0.012 | - |
| *Rake marks (VHA)* | 51 | 44 | 0.20 | 600 | 97 | 0.025 | - |
| *Age* | 73 | 58 | 0.20 | 300 | 174 | 3.106 | 0.51 |
| *Sex* | 93 | 75 | 0.10 | 500 | 255 | 0.071 | - |
| *Year* | 103 | 85 | 0.20 | 300 | 263 | 0.530 | 0.65 |

**Supplementary Table 2.** Year, number of samples collected, and date range of sample collection for respiratory exhalant samples collected by drone from North Atlantic right whales in Cape Cod Bay, Massachusetts, USA.

| **Year** | **Samples collected** | **Date range of sample collection** |
| --- | --- | --- |
| 2016 | 9 | March 23 – April 09 |
| 2017 | 14 | March 25 – April 27 |
| 2018 | 10 | March 28 – April 28 |
| 2019 | 2 | April 07 – April 14 |
| 2022 | 18 | March 15 – May 06 |
| 2023 | 28 | March 22 – April 27 |
| 2024 | 22 | April 08 – April 26 |

**SUPPLEMENTARY FIGURES**


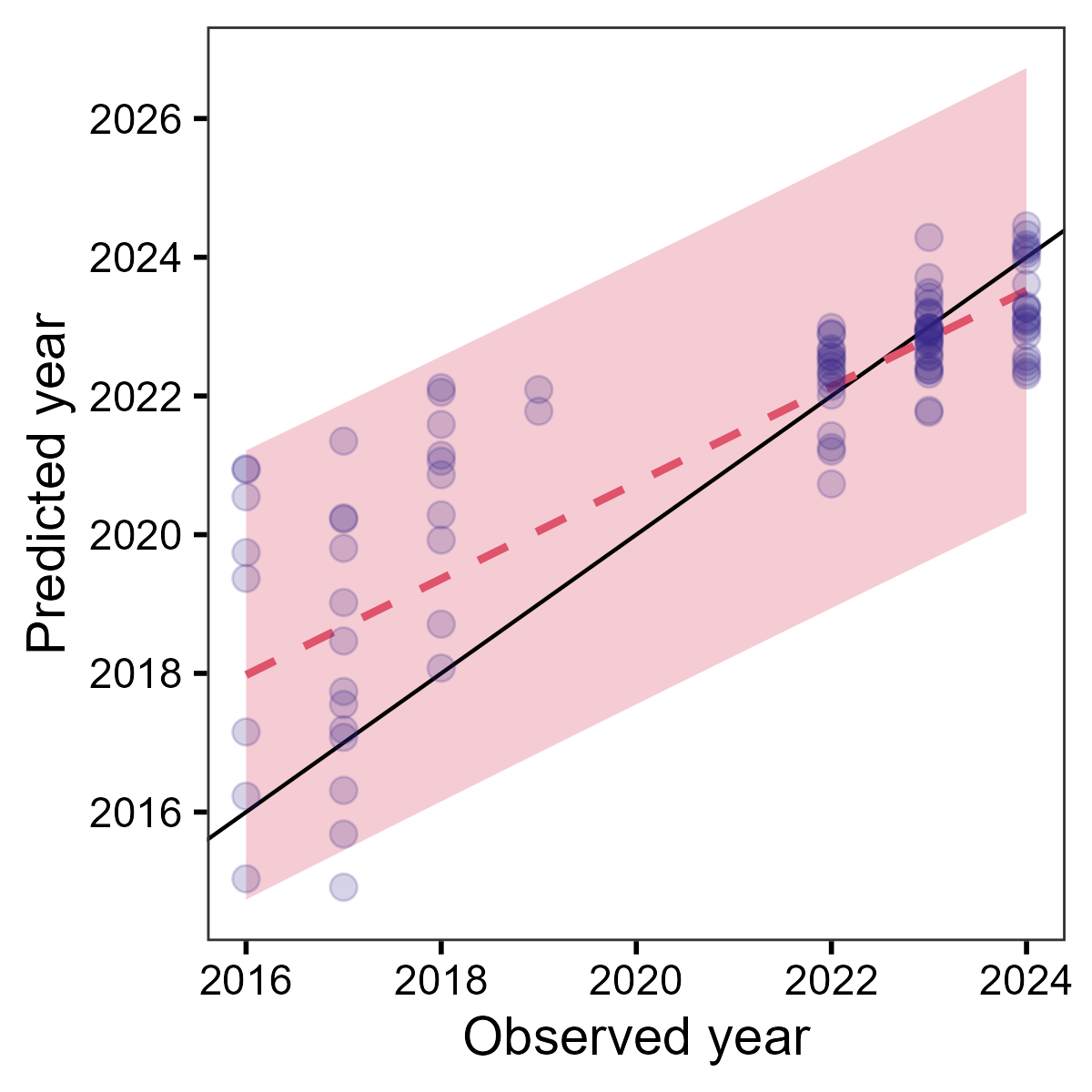


**Supplementary Figure 1.** Results of the elastic net regression for year when removing seawater-prevalent ASVs. Predictions were obtained using leave-one-out cross-validation. The dashed line and shaded area indicate the estimated relationship (mean and 95% prediction interval) between observations and elastic net predictions, and the black line represents the 1-1 diagonal.


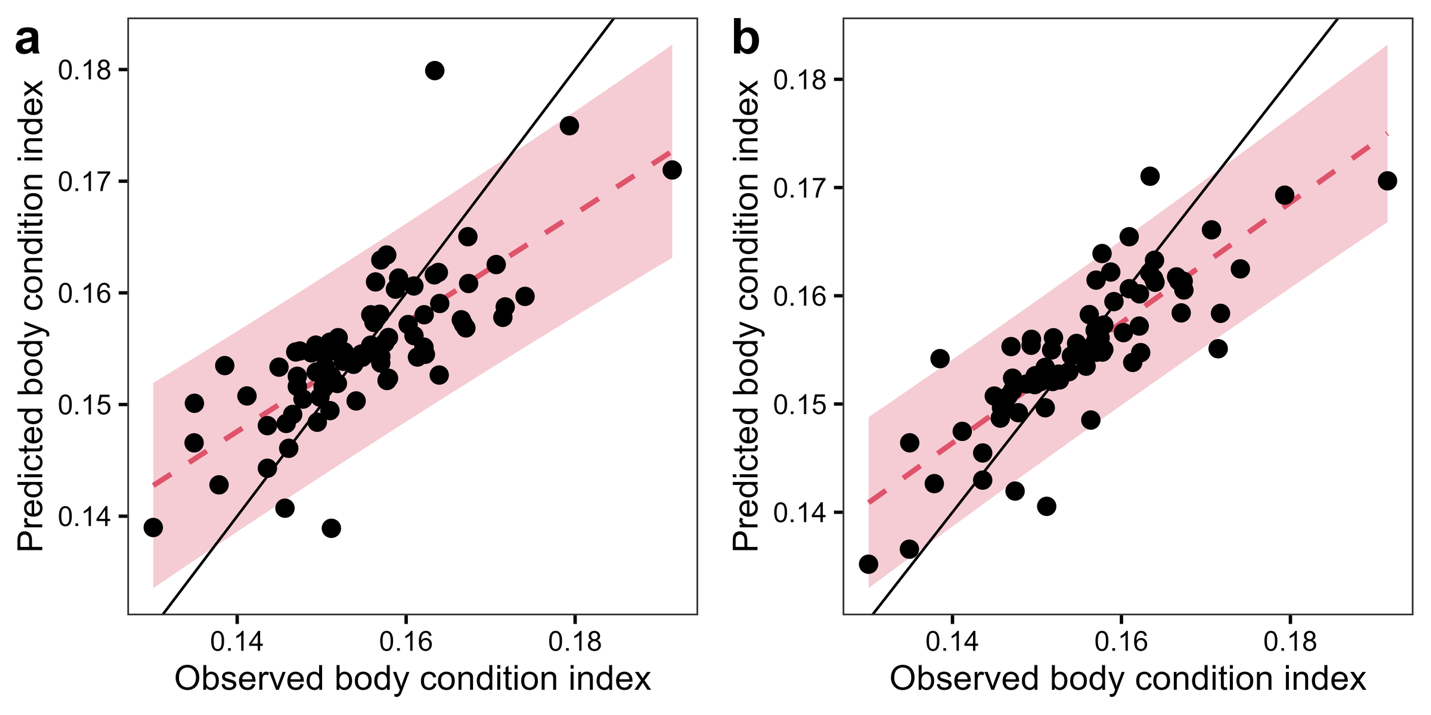


**Supplementary Figure 2.** Results of the elastic net regression for body condition index from photogrammetry when using a leave-one-individual-out cross-validation for prediction. In a, the pre-screening of putative explanatory variables was also repeated; in b, the initial set of putative explanatory variables was taken from the analysis presented in the main text. The dashed line and shaded area indicate the estimated relationship (mean and 95% prediction interval) between observations and elastic net predictions, and the black line represents the 1-1 diagonal.


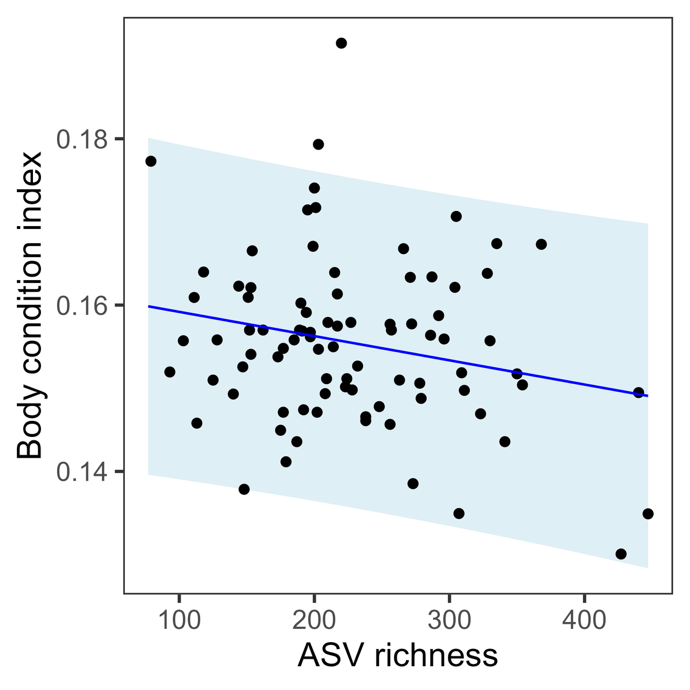


**Supplementary Figure 3.** Univariate association between ASV richness and body condition index. The line and shaded area represent the estimated mean and 95% prediction interval from a Gaussian linear model.

**SUPPLEMENTARY REFERENCES**

1. Durban JW, Moore MJ, Chiang G *et al.* Photogrammetry of blue whales with an unmanned hexacopter. *Mar Mamm Sci*. 2016;**32**:1510-15 <https://doi.org/10.1111/mms.12328>

2. Miller CA, Perryman WL, Best PB *et al.* Body shape changes associated with reproductive status, nutritive condition and growth in right whales *Eubalaena glacialis* and *E. australis*. *Mar Ecol Prog Ser*. 2012;**459**:135-56 <https://doi.org/10.3354/meps09675>

3. Pirotta E, Schick RS, Hamilton PK *et al.* Estimating the effects of stressors on the health, survival and reproduction of a critically endangered, long-lived species. *Oikos*. 2023;**2023** <https://doi.org/10.1111/oik.09801>

4. Pirotta E, Tyack PL, Durban JW *et al.* Decreasing body size is associated with reduced calving probability in critically endangered North Atlantic right whales. *R Soc Open Sci*. 2024;**11**:240050 <https://doi.org/10.1098/rsos.240050>

5. Pettis HM, Rolland RM, Hamilton PK *et al.* Visual health assessment of North Atlantic right whales (*Eubalaena glacialis*) using photographs. *Can J Zool*. 2004;**82**:8-19 <https://doi.org/https://doi.org/10.1139/z03-207>

6. Durban JW, Fearnbach H, Barrett-Lennard LG *et al.* Photogrammetry of killer whales using a small hexacopter launched at sea. *Journal of Unmanned Vehicle Systems*. 2015;**3**:131-35 <https://doi.org/https://doi.org/10.1139/juvs-2015-0020>

7. North Atlantic Right Whale Consortium. North Atlantic Right Whale Consortium Visual Health Assessment Database. v. 07/11/2024. Boston, MA, USA: Anderson Cabot Center for Ocean Life at the New England Aquarium.

8. Apprill A, Miller CA, Moore MJ *et al.* Extensive Core Microbiome in Drone-Captured Whale Blow Supports a Framework for Health Monitoring. *mSystems*. 2017;**2**:e00119-17 <https://doi.org/10.1128/mSystems.00119-17>

9. Apprill A, McNally S, Parsons R *et al.* Minor revision to V4 region SSU rRNA 806R gene primer greatly increases detection of SAR11 bacterioplankton. *Aquat Microb Ecol*. 2015;**75**:129-37 <https://doi.org/http://dx.doi.org/10.3354/ame01753>

10. Parada AE, Needham DM, Fuhrman JA. Every base matters: assessing small subunit rRNA primers for marine microbiomes with mock communities, time series and global field samples. *Environ. Microbiol*. 2016;**18**:1403-14 <https://doi.org/https://doi.org/10.1111/1462-2920.13023>

11. Callahan BJ, McMurdie PJ, Rosen MJ *et al.* DADA2: High-resolution sample inference from Illumina amplicon data. *Nature Methods*. 2016;**13**:581-83 <https://doi.org/10.1038/nmeth.3869>

12. Davis NM, Proctor DM, Holmes SP *et al.* Simple statistical identification and removal of contaminant sequences in marker-gene and metagenomics data. *Microbiome*. 2018;**6**:226 <https://doi.org/10.1186/s40168-018-0605-2>

13. Zou H, Hastie T. Regularization and variable selection via the elastic net. *J R Stat Soc B*. 2005;**67**:301-20 <https://doi.org/https://doi.org/10.1111/j.1467-9868.2005.00503.x>

14. Friedman J, Hastie T, Tibshirani R. Regularization Paths for Generalized Linear Models via Coordinate Descent. *J Stat Softw*. 2010;**33**:1-22 <https://doi.org/https://doi.org/10.18637/jss.v033.i01>

15. R Development Core Team. R: A language and environment for statistical computing [Computer software]. R Foundation for Statistical Computing. <https://www.R-project.org/>. 2020.

16. Fan JQ, Lv JC. Sure independence screening for ultrahigh dimensional feature space. *J R Stat Soc B*. 2008;**70**:849-83 <https://doi.org/https://doi.org/10.1111/j.1467-9868.2008.00674.x>

17. Yoon G, Zheng YN, Zhang Z *et al.* Ultra-high dimensional variable selection with application to normative aging study: DNA methylation and metabolic syndrome. *BMC Bioinformatics*. 2017;**18** <https://doi.org/https://doi.org/10.1186/s12859-017-1568-1>
